# Intraoperative Diagnosis of Central Nervous System Tumors: Challenges, Errors, Lessons Learned, and the Surgeon’s Perspective

**DOI:** 10.7759/cureus.17823

**Published:** 2021-09-08

**Authors:** Yookarin Khonglah, Bifica Sofia Lyngdoh, Arindom Kakati, Jaya Mishra, Mostafa Muhammad Al Aman, Pranjal Phukan

**Affiliations:** 1 Department of Pathology, North Eastern Indira Gandhi Regional Institute of Health & Medical Sciences, Shillong, IND; 2 Department of Neurosurgery, North Eastern Indira Gandhi Regional Institute of Health & Medical Sciences, Shillong, IND; 3 Department of Radiology, North Eastern Indira Gandhi Regional Institute of Health & Medical Sciences, Shillong, IND

**Keywords:** cns tumor, intraoperative, challenges, errors, crush smears, central nervous system tumors

## Abstract

Background: Intraoperative crush smear is an adjuvant in diagnosing central nervous system (CNS) lesions on tissue sent for frozen section. Besides rapid decision-making, it also ensures that minimum injury is caused to the normal brain structures surrounding the intracranial neoplasm. A rapid intraoperative diagnosis helps the surgeon in planning the appropriate surgery.

Objective: Our objective is to review all the discordant cases between intraoperative and histopathological diagnosis and also to study the crush smear slides for morphological clues that could have been helpful in minimizing such errors, especially for an inexperienced neuropathologist/general pathologist. The surgeon’s perspective on the impact of these errors on management is also discussed.

Method: A prospective study of six years from 2013 to 2019 was conducted. Crush smears were made and stained with rapid hematoxylin and eosin (H&E). The rest of the tissue was processed for permanent tissue sections. Slides in which there was discordance between the intraoperative and permanent paraffin sections were reviewed to ascertain the reasons thereof.

Results: A total of 81 specimens of CNS tumors were sent for intraoperative consultation. Out of these, discordance was seen in 13 (16%) cases.

Conclusion: To minimize diagnostic errors, it is important to do regular analyses of the misinterpreted cases. Knowledge of the pre-operative radiological differential diagnosis is mandatory. Discussion with the surgeon regarding the clinical impact of the errors made will give a clearer picture to the pathologists regarding clinically relevant reporting during intraoperative consultation.

## Introduction

Brain tumors are a diverse group of neoplasms originating from intracranial tissues and the meninges [1]. Intraoperative crush smear is an adjuvant in the diagnosis of central nervous system (CNS) tumors sent for frozen section. A rapid intraoperative diagnosis is achieved by doing crush preparation and this can help the surgeon to plan the appropriate surgery. The innately soft nature of brain tissue produces poor quality frozen sections. With the advent of stereotactic biopsy, the role of crush smear/squash preparation has assumed more importance as only a small amount of tissue is used and it also ensures minimum injury is caused to adjacent normal brain tissue [2,3]. Misdiagnosing on crush smears may have a significant impact on the management of the patients.

Our objective is to review all the discordant cases between intraoperative diagnosis and histopathological diagnosis and also to study the crush smear slides for morphological clues or any other information that could have been helpful in minimizing such errors for an inexperienced neuropathologist/general pathologist. The surgeon’s perspective on pathological errors made and their impact on management is also discussed.

## Materials and methods

A prospective study of six years from August 2013 to August 2019 was conducted in the Department of Pathology of our institute. Approval for our study was issued by the Institution Ethics Committee, North Eastern Indira Gandhi Regional Institute of Health & Medical Sciences, Shillong (NEIGRIHMS).

Crush smears were done on all specimens sent as intraoperative consultation. About 1 mm of the tissue, each from areas of different color and consistency is chosen. The tissues were gently crushed with minimum pressure and drawn towards the edge of the slide evenly with another clean slide. Two to three slides were fixed in 95% ethanol for hematoxylin and eosin (H&E) stain and the rest two to three slides were air-dried for Giemsa staining. Slides are stained with rapid H&E and Giemsa stains. All slides were examined before an intraoperative diagnosis is made. A provisional diagnosis with clinico-radiological correlation was given. The final diagnosis was given on paraffin-embedded histopathological sections. Immunohistochemistry (IHC) was done in tumors that required further subtyping.

Slides in which there was discordance between the intraoperative and permanent paraffin sections were later reviewed to look for the reasons for the discrepancy.

## Results

During the period of study, a total of 81 cases of CNS tumors were sent for intraoperative consultation. There was discordance in 13 (16 %) out of 81 cases. Therefore the diagnostic accuracy of crush smear in our study was 84% (68/81). The cases showing discordance are summarized in Table 1, stating the pre-operative diagnosis, intraoperative diagnosis, final H&E diagnosis, reasons for discordance, lessons learned, and the surgeon’s remarks/clinical impact.

**Table 1:** Details of all the cases including a summary of errors, lesson learned, and surgeon’s remarks SOL: space-occupying lesion; NOS: not otherwise specified; DLBCL: diffuse large B-cell lymphoma; PNET: primitive neuroectodermal tumor; SEGA: subependymal giant cell astrocytoma

Case no.	Age/Sex	Preoperative clinico-radiological diagnosis	Intraoperative crush smear diagnosis	Permanent H&E diagnosis	Reasons forepancy	Lessons learned	Surgeons remarks (clinical impact)
1.	15/F	Parieto-occipital lobe SOL ?Low-grade glioma	Pilocytic astrocytoma	Ependymoma, WHO Grade II	Rosenthal fibres+. Perivascular rosettes were overlooked. Cells were slender with thin and delicate processes.	Excessive force while making crush smears may lead to morphological changes (slender cells) and is to be avoided	Both need to be excised totally
2.	14/M	Left frontal SOL ?Hemangiopericytoma	Malignancy cannot be ruled out	Anaplastic Oligoastrocytoma NOS WHO Grade III	Inadequate tissue with few atypical cells	Repeat biopsy may have been helpful	Both require maximal safe excision
3.	55/F	Right midline parafalcine mass	Meningioma	Hemangiopericytoma WHO Grade II	Tissue not crushable, smear showed presence of spindle cells.	For firm tissues. Frozen section is advised	Both need total excision
4.	45/M	L3-L4 intradural extramedullary spinal tumor ?Myxopapillary Ependymoma ?Schwannoma	Myxopapillary ependymoma	Schwannoma with myxoid degeneration	Loosely textured stellate shaped cells, in a myxoid background	Sampling bias due to secondary changes in tumors may result in varied morphological changes	Both require surgical excision
5.	50/M	Frontal lobe SOL ?Ependymoma	Ependymoma	Transitional meningioma WHO Grade I	Sampling error: loose areas of the tumor lead to interpretation of perivascular rosettes	Tissue should be sampled from different looking areas	Both need total excision
6.	12/F	Posterior fossa brain tumor ?Pilocytic astrocytoma ?Medulloblastoma	Astrocytoma-grade II cannot rule out grade III	Medulloblastoma, NOS WHO Grade IV	Inability to recognize the round blastic immature cells and missed the mitotic figure	Careful examination of the smears will help pick clues to a correct diagnosis despite the time constraints	Both need maximal possible excision
7.	55/F	?Demyelinating lesion – B/L cerebral hemisphere	Inflammatory lesion. Cannot rule out malignancy	Diffuse large B-cell lymphoma (DLBCL)	Steroid-induced tumor regression	Imprint smear combine with crush smears may increase the sensitivity of diagnosing lymphoma in such a case	In lymphoma, there is not much use of surgery except for establishing a diagnosis by biopsy
8.	30/F	Right intraventricular right parietal tumor 1. Atypical meningioma 2. PNET 3. Choroid plexus papilloma	Atypical meningioma	Anaplastic meningioma WHO Grade III	Sampling error. Infrequent number of mitosis. No necrosis seen	Tissue should be sampled from different looking areas	Surgical treatment same
9.	25/F	Left frontal SOL ?Oligodendroglioma	High grade Oligodendroglioma	Glioblastoma NOS WHO Grade IV	Absence of hallmark mitotic figures. Subjective interpretation of pleomorphism and cellularity	On intraoperative consultations, differentiating low-grade from high-grade gliomas is enough for the surgeon	Both require maximal possible excision followed by multimodality treatment
10	7/M	Pineal region tumor 1. Germinoma 2. Pineal tumor	Pineocytoma	Pineoblastoma WHO Grade IV	Sampling error: not enough clinical correlation	Request for deeper representative sample and knowledge of normal brain cytology	Gross total resection is the goal in pineocytoma vs multimodality treatment in pineoblastoma.(Discussed in text)
11	13/F	Intraventricular SOL 1. Neurocytoma 2. Ependymoma 3. Choroid plexus papilloma	Ependymoma	Subependymal giant cell astrocytoma (SEGA) WHO Grade I	Misinterpretation of cells arranged peri-vascularly as rosettes.	Careful screening for features like nuclear size and multinucleation	Surgical excision when required is the treatment.
12.	52/M	Left cerebellum SOL	Low-grade glioma	Reactive gliosis. A repeat biopsy showed hemangioblastoma	Misinterpretation of the cellularity of smear as hypercellular due to the interspersed inflammatory cells	Always look for monotonous cells, atypia. If in doubt, ask for another biopsy sample especially when there is no correlation with the clinical and radiological findings	Repeat biopsy
13	30/F	Intramedullary spinal cord lesion	Low-grade Glioma	Reactive gliosis No record of a repeat biopsy			Repeat biopsy

## Discussion

Tumors of the CNS are not very common; however, the incidence is increasing rapidly. The worldwide incidence of primary malignant brain tumors in 2008 was 3.8 per 100,000 in males and 3.1 per 100,000 in females [4]. According to our biopsy statistics, the incidence of CNS tumors is 5.6%. This is quite high in a developing country like ours [5], and similar to the incidence of brain tumors as seen in developed countries [6]. However, as the study represents only the surgically treated CNS tumors, a selection bias invariably exists in our study.

Soft, friable tissues can be easily made into smears, yielding good cellularity [7]. The majority of gliomas belong to this category, including medulloblastomas and metastatic carcinomas. These lesions posed few diagnostic problems as the cell yield was good. On the other hand, in the case of firm tissue, such as in meningiomas and schwannomas, smears were difficult to make and so did not yield good cellularity.

The possible reasons for the discordance between crush smears and histological sections are discussed case-wise, based on the morphological clues that might have been overlooked during the intraoperative diagnosis and correlated clinico-radiologically. The clinical impact of each of these errors is also discussed including the surgeon’s perspective.

Cases and lessons learned

Case 1: Parieto-occipital Lobe Space-Occupying Lesion (SOL), 15 Years/F

A case of ependymoma was diagnosed as pilocytic astrocytoma (PA) on crush cytology. On review of the slides, it was realized that Rosenthal fibers were seen and the perivascular rosettes were distorted and, therefore, overlooked leading to a misinterpretation. The cells in PA are slender, bipolar, and stellate in shape with long, thin, and delicate processes. The cells in ependymoma show extreme fibrillarity and the nuclei are round to oval, 'carrot-shaped' with dense nuclear chromatin showing slight nuclear pleomorphism [8]. Sometimes, when an excessive shearing force is applied during the crushing process, the cells will appear thinned out and eventually get separated from the vessels. This probably happened in our case and, as a result, the perivascular rosettes were not appreciated as required for the diagnosis of ependymoma. Teo, et al. have also described similar findings in their study [9].

Lesson learned:Excessive force while making crush smears is to be avoided.

Case 2: Left Frontal SOL, 14 Years/M

This case had a radiological diagnosis of hemangiopericytoma and the tissue in crush was inadequate; we, however, saw some atypical cells and reported the crush smear as malignancy could not be excluded. In this case we could at least inform the surgeon that it was a neoplastic lesion.

Lesson learned: Repeat biopsy may have been helpful

Case 3: Right Midline Parafalcine Mass, 55 Years/F

One case of hemangiopericytoma was misdiagnosed as meningioma. On examining the smear only a few spindle cells were seen since the tissue was not crushable. The main aim for intraoperative consultation, in this case, was to exclude a high-grade tumor, which we were able to.

Lesson learned: For firm tissues, frozen section is advised.

Case 4: L3-L4 Intradural Extramedullary Spinal Tumor, 45 Years/M

**Figure 1:**
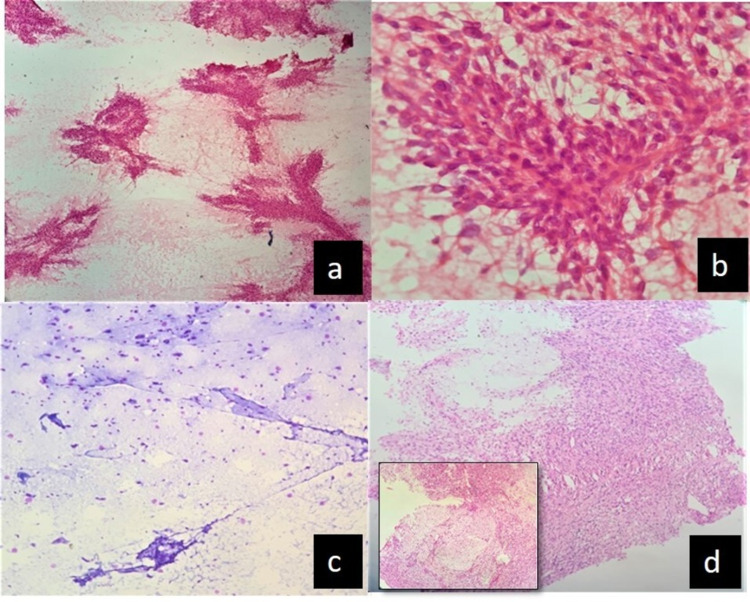
In case 4, schwannoma reported on crush smear as myxopapillary ependymoma (a) and (b): Crush smears show cellular tumor cells radiating from the vessels reported as radiating perivascular cells (H&E, x100, x400) (c) Myxoid-like areas (H&E, x100) (d) Sections showing predominance of Antoni A component with inset showing focal myxoid change (H&E, x 100)

One case was interpreted as myxopapillary ependymoma based on the clinico-radiological picture. This case on formalin-fixed paraffin-embedded sections was diagnosed as schwannoma with myxoid changes. Secondary myxoid changes may occur in schwannoma and lead to an error in diagnosis. Schwannoma is an important differential of myxopapillary ependymoma. However, the classic biphasic pattern of schwannoma (Antoni A and B patterns), and the occasional presence of Verocay bodies are distinctive features that are generally not encountered in the myxopapillary ependymoma. The true ependymal rosettes and perivascular pseudorosettes that mark ependymoma histologically are generally absent or not well-formed in the myxopapillary ependymoma [10]. Our case had significant Antoni B areas containing loosely textured stellate-shaped cells, which is known to mimic glial neoplasm. Also, the myxoid changes seen in the tumor were mistaken for the myxohyaline material seen in myxopapillary ependymoma. Figure 1 a-d.

Lesson learned: Sampling bias due to secondary changes in tumors results in varied morphological changes that may lead to multiple differential diagnoses for the pathologist and should be interpreted cautiously.

Case 5: Frontal Lobe SOL, 50 Years/M

A case of transitional meningioma was misinterpreted as ependymoma. When the slide was reviewed, we felt it could be due to a sampling error that picked up loose areas of the tumor leading to a mistaken interpretation of perivascular rosettes.

Lesson learned: Tissue should be sampled from different-looking areas.

Case 6: Posterior Fossa Tumor, 12 Years /F

One case of medulloblastoma was reported as grade II astrocytoma (cannot rule out grade III astrocytoma) on the crush smears. The radiological diagnosis was ?pilocytic astrocytoma vs medulloblastoma. On review of smears and close observation of cell morphology, they simulated the round blastic immature cells seen in medulloblastoma cases that were not recognized during intraoperative diagnosis. Apart from the high cellularity and vascularity of the smears, which made us report the possibility of grade III astrocytoma, we also detected a mitotic figure on review. The pressure of diagnosis within a limited time in an intraoperative consultation is one major factor for an incorrect interpretation. We had missed the mitotic figure and, according to Joseph [8], a single mitotic figure on a crush smear warrants a diagnosis of a high-grade lesion. Kishore, et al. [11] and Kini, et al. [12], and others have reported 100% accuracy in diagnosing medulloblastomas on smear cytology.

Lesson learned:Sometimes radiological diagnosis may be ambiguous and, in such cases, careful examination of the smears and experience will help pick clues to a correct diagnosis despite the time constraints.

Case 7: ?Demyelinating Lesion - Bilateral Cerebral Hemispheres, 55 Years/M

**Figure 2:**
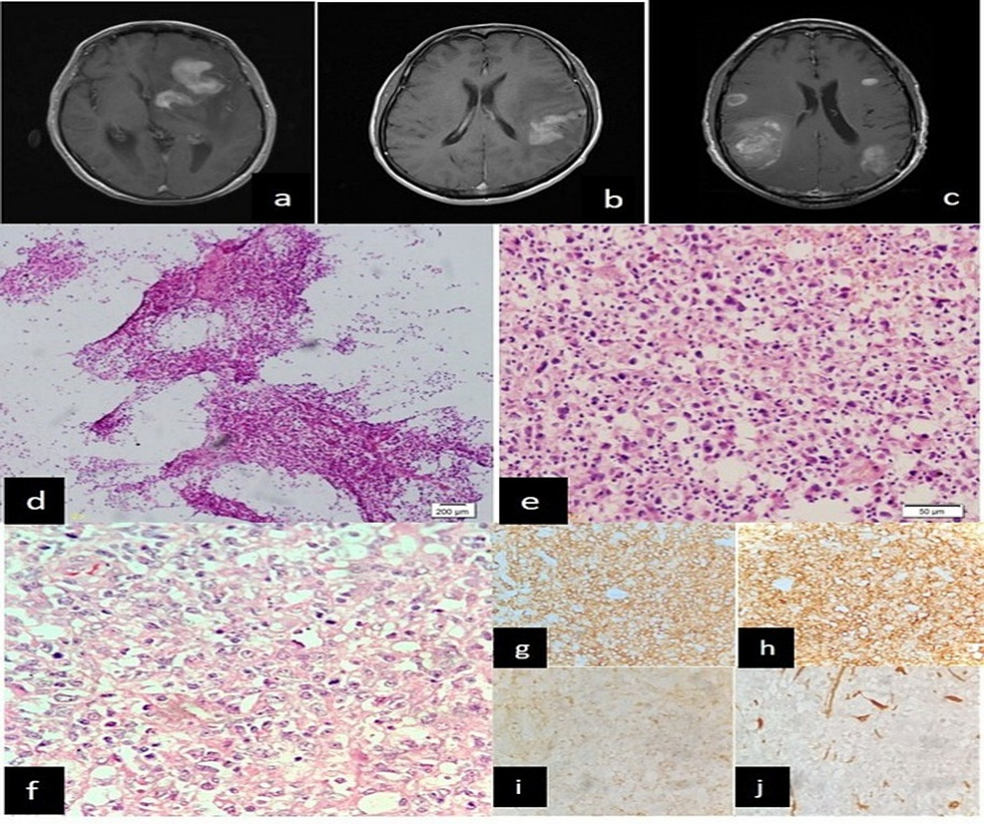
In case 7, lymphoma diagnosed radiologically as demyelination, non-diagnostic on crush smear (a) Post-contrast T1 axial images show multiple intensely, solidly contrast-enhancing masses involving the left basal ganglia and frontal white matter (b) Left parietal lobe and (c) bilateral fronto-parietal parenchyma (d and e) Crush smears showing mainly inflammatory, degenerated, and few atypical cells (H&E x100, x400) (f) Sections showing monotonous population of cells (g) CD45 positive (h) CD20 positive (i) CD3 negative in tumor cells (j) GFAP negative in tumor cells (IHC stains x400) CD: cluster of differentiation; GFAP: glial fibrillary acidic protein; IHC: immunohistochemistry; H&E: hematoxylin and eosin

This was a patient with a radiological impression of tumefactive demyelination based on which the patient was started on steroids. He was not improving clinically and a repeat scan showed multiple bilateral lesions mandating a biopsy, so tissue was received for intraoperative consultation. The crush smear showed numerous inflammatory cells along with the presence of necrosis and occasional atypical degenerated cells. A deeper biopsy revealed more inflammatory cells. It was reported as an inflammatory lesion but the possibility of malignancy could not be ruled out. Postoperatively, on discussion with the multi-disciplinary team, other differential diagnoses were included: lymphoma vs metastasis (epithelial/malignant melanoma) vs anaplastic oligodendroglioma. Immunostaining was done for cytokeratin, glial fibrillary acidic protein (GFAP), human melanoma black-45 (HMB-45), cluster of differentiation (CD) 45, CD3, and CD 20. IHC was positive for human leukocyte common antigen (LCA), B-cells, and T-cells (in the reactive population of cells). It was then diagnosed as primary CNS lymphoma (diffuse large B-cell lymphoma). Retrospectively, the main reason for the inconclusive diagnosis on crush was due to steroid-induced tumor regression in lymphoma, as a result, abundant degenerated neutrophils, macrophages were obscuring the underlying lymphoma. In lymphoma, intraoperative diagnosis is required only to get enough tissue for establishing a diagnosis by biopsy as there is not much role of surgery. The crush smear slides were reviewed, but the result remained inconclusive. Figure 2 a-j.

Lesson learned: Taking an imprint smear along with a crush may help in better visualization of the cells and may increase the sensitivity of detecting lymphoma cells.

Case 8: Right Intraventricular Right Parietal Tumor, 30 Years /F

Another case of anaplastic meningioma was diagnosed as atypical meningioma. On cytology and frozen section, atypical meningioma can be diagnosed based on high cellularity, moderate nuclear pleomorphism, and the presence of scattered mitotic figures [13]. This case of anaplastic meningioma was diagnosed as atypical meningioma because only infrequent mitotic figures were noted. Ali, et al. state that it is usually difficult to grade meningiomas on cytology and suggests a differentiation between atypical and anaplastic meningioma by features such as necrosis and mitosis [14]. Savargoankar, et al. in their series of 103 cases have reported that most discrepancies were due to failure to identify atypia in meningioma [15].

Lesson learned: Features like necrosis and mitosis, which help to grade meningiomas, may not be seen in the slide. So, taking samples from multiple different-looking areas with different consistencies will help get the most out of the sample.

Case 9: Left Frontal SOL, 25 Years/F

A case of glioblastoma was misdiagnosed as high-grade oligodendroglioma on crush smears. On review of the slides, the reason for misinterpreting the diagnosis may be due to the loss of hallmark mitotic figures during crush smear preparation. Acharya, et al. showed similar findings in their study and reported that we rely on subjective analysis of the degree of cellularity and pleomorphism which may lead to discrepancies in some high-grade gliomas [16]. The nucleus in anaplastic oligodendroglioma is usually round to oval whereas in glioblastoma they are more elongated [9].

Lesson learned: On intraoperative consultations, confirming the presence of neoplastic tissue and differentiating low-grade from high-grade glioma is enough for the surgeon.

Case 10: Pineal Lesion, 7 Years/M

A case of pineoblastoma was diagnosed as pineocytoma since we did not find any atypical features, mitotic figures, or necrosis in the smears. On review of the slide, we concluded that the tissue we saw on crush could have been a bit of pineal gland which cytologically cannot be easily differentiated from pineocytoma [17].

**Figure 3:**
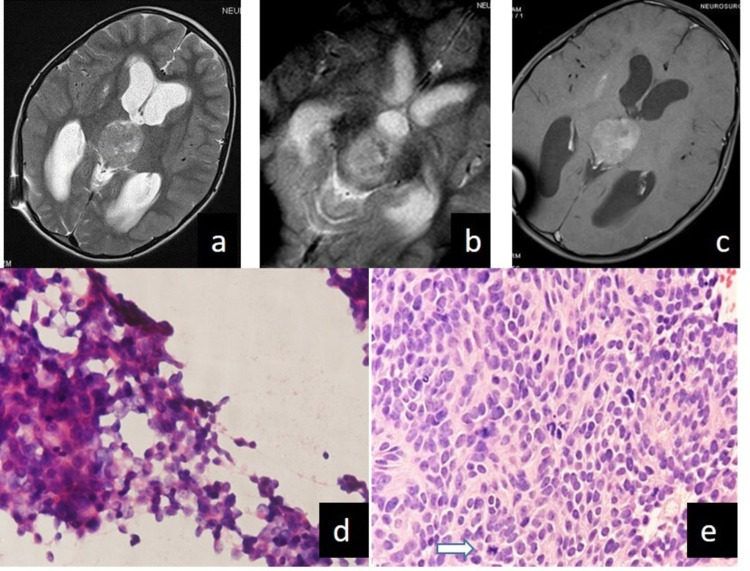
In case 10, pineoblastoma reported on crush smear as pineocytoma (a) A well-circumscribed mass is seen on T2WI originating from pineal gland compressing the superior colliculus and aqueduct with resulting ventriculomegaly (b) Tumoral calcification is seen at the peripheral of the lesion on GRE images (c) Contrast study reveal marked enhancement of the lesion with no CSF spread (d) Uniform population of tumor cells resembling normal pineal gland (H&E x 400) (e) Section showing densely packed sheets of anaplastic cells with large hyperchromatic nuclei and minimal cytoplasm. White arrow marks mitotic figure.(H&E x400) GRE: gradient recalled echo; CSF: cerebrospinal fluid; H&E: hematoxylin and eosin

Lesson learned:When radiology is not conclusive and the patient is a young child, we should have had a high index of suspicion for a pineoblastoma since pineocytoma in a child is extremely rare. This should have led us to ask for a deeper representative sample. Pre-operative discussion with the multidisciplinary team is mandated for every case and such misdiagnosis could have been averted. The radiological images were reviewed and imaging findings favored pineoblastoma depicted in Figure 3.

Surgeons Remarks on Clinical Impact: In pineocytoma, gross total excision is the treatment of choice. So if diagnosed intraoperatively, extra effort is made to excise it completely as there is no role of radiation and chemotherapy. If total excision is not possible, then follow-up with serial scans, re-exploration, and excision is the only option, which tends to have higher morbidity. On the other hand, pineoblastomas, since they are very aggressive and frequently metastasize, the treatment is maximal surgical excision possible followed by chemotherapy and radiotherapy. Hence, there is no need to remove every piece of tumor since invariably they recur [18]. However, the most important fact that we need to know during pineal region surgery is whether the tumor is a germinoma or not as they are exquisitely radiosensitive and surgery has a very limited role.

Case 11: Intraventricular SOL, 13 Years/F

One case of subependymal giant cell astrocytoma (SEGA) was misdiagnosed as ependymoma on crush cytology. On review of the slide, it was seen that the tumor cells were arranged perivascularly as seen in ependymomas. However, on a closer look at the cells, it is seen that the individual cells are bigger in size compared to ependymoma cells and also had an abundant eosinophilic cytoplasm in a fibrillary background. Also seen were occasional bi and multinucleated cells. Similar findings were noted by Takei H, et al. [19]. Correlating with the age, location of the tumor, and the radiological findings our diagnosis was favoring ependymoma.

Lesson learned: When the differential diagnosis is close, careful examination for features that will favor one diagnosis over the other is mandated, in this case, nuclear size and multinucleation.

Case 12: Left Cerebellum SOL, 52 Years/M & Case 13: Intramedullary Spinal Cord Lesion, 30 Years/F

**Figure 4:**
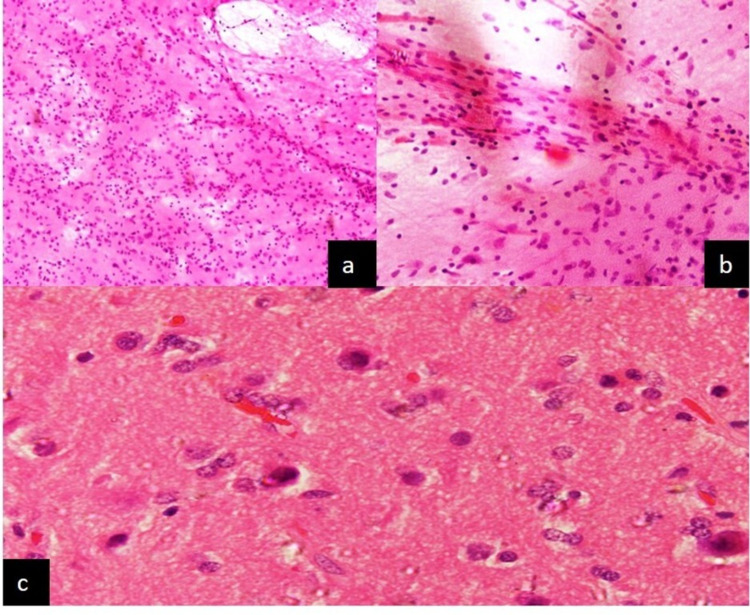
In case 12, hemangioblastoma reported on crushsmear as low-grade glioma (a) Smears showing increase cellularity (H&E x100) (b) Hypercellularity was due to inflammation (H&E x 400) (c) Sections show features of Reactive gliosis (H&E x 400) H&E: hematoxylin and eosin

In our study, case 12 (Figure 4 a-c) and case 13 of reactive gliosis were misinterpreted as low-grade glioma. On review of the slide, we found that because of the inflammation the smears looked a little hypercellular compared to normal brain tissue. However, on review and careful examination on higher magnification, it was seen that there was a mixture of cells giving it a heterogenous appearance unlike in a neoplastic process where we can see only a single type of cell population with pleomorphism [8]. The reactive astrocytes did not show significant nuclear atypia.

Lesson learned: As nearly every injury to the CNS induces gliosis (“Evil often lurks in a reactive picture”), therefore it is always advisable to request a second sample in cases where the radiological findings do not correlate with the pathological findings. Reactive astrocytes lack nuclear atypia and high cell density, have long, tapering, starlike (astro) cytoplasmic processes, as opposed to the short and irregular blunted processes in neoplastic astrocytes. Neoplastic astrocytes have coarse chromatin with convoluted nuclear membrane [8].

Challenges and how to overcome them

We undertook this study believing that our errors could have led to mismanagement but realized that in most cases (10/13) we were able to guide the surgeons by confirming adequate sampling of neoplastic tissue. The challenges faced are the time constraints, anxiousness to not make a mistake (which invariably leads to one!), or lack of confidence due to inexperience. Looking at the clinical impact of our errors (Table 1), one should feel encouraged that the errors were not damaging and be less anxious during intraoperative consultations, which may lead to better reporting. These challenges can be overcome by prior knowledge of the clinico-radiologic differential diagnoses, discussion with the surgeons and radiologists in a multi-disciplinary team pre-operatively. We have to understand what the surgeons expect from a pathologist during intraoperative consultations: to confirm the adequacy of the sample, confirm tissue as neoplastic, to look for any discrepancy between the radiological diagnosis and the intraoperative diagnosis, which could be due to a sampling error or variations within the tumor leading to sampling bias, which could lead to a different diagnosis from what was expected.

Surgeon’s perspective

The role of intraoperative diagnosis for a neurosurgeon is first, whether adequate (correct) tissue has been sampled as it becomes a herculean task to go in again for repeat biopsy; second is the nature of the lesion - whether inflammatory or neoplastic; benign or malignant. In case of suspected infectious lesions, a microbiological sample is mandated. The aim in benign lesions is for complete excision if anatomically and physiologically feasible. In malignant lesions, it does not really matter intraoperatively since the idea is maximum safe decompression as postoperatively patients would invariably require radiation and chemotherapy [20-23].

What the surgeons are apprehensive about is not removing something that they should have removed (benign lesion in the non-eloquent region) and removing something they should not have (inflammatory lesions or radio and chemosensitive tumors like lymphomas and germinomas) [20-23].

Limitations of the study

The caseload is extremely low; however, this paper is written to encourage the young pathologists and as a reminder of the basics in the interpretation of intraoperative crush smears of CNS lesions and especially to understand their role and the clinical impact their diagnosis could make on the surgery.

## Conclusions

In an intraoperative consultation, the time constraints and the pressure of calls from the operating theater for a rapid diagnosis may sometimes rattle the nascent pathologists into giving an erroneous diagnosis. Our aim, as pathologists, is to give the correct or at least the closest diagnosis that will not hamper the management of the patient. We can fulfill this by understanding the challenges and how to overcome them as discussed. Intraoperative cytological evaluation constitutes an effective diagnostic modality, particularly on small samples with the caveat that its limitations must be clearly understood by both pathologists and neurosurgeons.
